# *Pneumocystis* Infection in Pregnant Women: A Scoping Review

**DOI:** 10.3390/jof11040327

**Published:** 2025-04-20

**Authors:** Irene Calderón-Baturone, Rocío Salsoso, Elena Charpentier, Yaxsier de Armas, Pilar Guadix, Rubén Morilla, Enrique J. Calderón, Vicente Friaza

**Affiliations:** 1 Programa de Doctorado en Ciencias de la Salud, Universidad de Sevilla, 41009 Seville, Spain; irecalbat@gmail.com; 2 Instituto de Biomedicina de Sevilla, Hospital Universitario Virgen del Rocío, Consejo Superior de Investigaciones Científicas/Universidad de Sevilla, 41013 Seville, Spain; elenacharpentier1@gmail.com (E.C.); rmorilla2@us.es (R.M.); vfriaza-ibis@us.es (V.F.); 3 Departamento de Medicina, Hospital Universitario Virgen del Rocío, Facultad de Medicina, Universidad de Sevilla, 41009 Seville, Spain; 4 Departments of Clinical Microbiology Diagnostic and Pathology, Hospital Center of Institute of Tropical Medicine “Pedro Kourí”, Havana 11400, Cuba; yaxsier2017@gmail.com; 5 Departamento de Microbiología y Patología, Instituto de Patología Infecciosa y Experimental “Francisco Ruiz Sánchez”, Guadalajara 44100, Mexico; 6 Obstetrics and Gynecology Service, Virgen Macarena University Hospital, School of Medicine, University of Seville, 41009 Seville, Spain; pilarguadix@gmail.com; 7 Centro de Investigación Biomédica en Red de Epidemiología y Salud Pública, 28029 Madrid, Spain; 8 Department of Nursing, Faculty of Nursing, Physiotherapy and Podiatry, Universidad de Sevilla, 41015 Seville, Spain

**Keywords:** *Pneumocystis*, pregnancy, colonization, pneumonia, scoping review

## Abstract

*Pneumocystis jirovecii* is an opportunistic fungus that causes severe pneumonia in immunosuppressed individuals. While *Pneumocystis* colonization, a subclinical form of infection, has been studied in different populations, its implications during pregnancy remain poorly understood. Given the immune modulation of pregnancy, maternal colonization or infection may contribute to vertical transmission and neonatal respiratory complications. This scoping review aims to map the existing evidence on *Pneumocystis* colonization/infection during pregnancy, identifying knowledge gaps, prevalence, risk factors, and potential neonatal outcomes. A systematic literature search was conducted in three databases following PRISMA-ScR guidelines. A total of 26 studies were included, covering *Pneumocystis* pneumonia cases (n = 19) and *Pneumocystis* colonization (n = 7). The review found that most *Pneumocystis* pneumonia cases in pregnant women were associated with HIV before antiretroviral therapy. More recent cases were related to hematologic malignancies. *Pneumocystis* colonization rates varied widely (5.4–46.5%). Evidence of vertical transmission was observed, but neonatal impact remains underexplored. This review highlights the need for HIV screening in pregnant women and the need to include *Pneumocystis* in the diagnosis of pregnant women with pneumonia. Increased awareness and research on *Pneumocystis* in pregnancy are necessary to improve maternal and neonatal outcomes. Future studies should focus on vertical transmission and neonatal respiratory health.

## 1. Introduction

*Pneumocystis jirovecii*, an opportunistic fungus, is a well-recognized pathogen that causes severe pneumonia (*Pneumocystis* pneumonia, PCP) in immunosuppressed individuals, particularly those with HIV/AIDS or who are undergoing immunosuppressive therapy [1]. However, *P. jirovecii* has a well-documented ability to infect individuals without causing overt pneumonia, a phenomenon referred to as colonization or carriage of *Pneumocystis* [2]. This subclinical form of infection involves the presence of the organism in the respiratory tract without significant symptoms or signs of active disease [2].

Colonization has been described in various populations, including immunosuppressed individuals such as those with HIV, cancer, or organ transplants, as well as in patients who receive corticosteroids or other immunosuppressive therapies [2,3]. Notably, *Pneumocystis* colonization has also been detected in seemingly healthy individuals, particularly among the elderly, infants, and patients with autoimmune and inflammatory diseases, populations characterized by immune modulation or immaturity [4,5,6]. The importance of colonization lies in its potential to act not only as a reservoir for disease transmission or as a possible transition period before PCP, but also as a trigger for inflammatory processes in the host, which may exacerbate underlying respiratory conditions or increase susceptibility to secondary infections [7].

However, its implications in pregnancy, a unique state of immune modulation, remain underexplored. Pregnancy is characterized by a delicate balance of immune adaptation to tolerate the fetus while maintaining defense against infections, creating a potentially vulnerable window for opportunistic pathogens such as *Pneumocystis* to thrive [8].

*P. jirovecii* has been identified in the placenta and lung tissue of fetuses that died in utero, showing its capacity for vertical transplacental transmission in humans [9]. Therefore, maternal colonization or infection could result in vertical transmission in newborns, with increasing evidence suggesting an association between exposure to *Pneumocystis* and neonatal respiratory complications such as respiratory distress syndrome (RDS) and bronchopulmonary dysplasia (BPD), conditions that contribute significantly to neonatal morbidity and mortality [10,11].

Despite these data, a comprehensive synthesis of the literature is lacking, leaving critical gaps in understanding the epidemiology, clinical presentation, and management of *Pneumocystis* infection or colonization during pregnancy.

This scoping review aims to map the existing evidence on *Pneumocystis* infection or colonization during pregnancy, providing a detailed overview of the prevalence, risk factors, diagnostic challenges, and outcomes reported in the literature. The review adopts the Arksey and O’Malley framework for scoping studies, which emphasizes a systematic approach to identifying and analyzing the breadth of available evidence [12]. In doing so, this review seeks to identify research gaps, inform clinical practice, and propose directions for future research on this critical yet under-represented topic.

## 2. Materials and Methods

The scoping review methodology was used for this study based on the framework proposed by Arksey and O’Malley and the Preferred Reporting Items for Systematic Reviews and Meta-Analyses (PRISMA) Extension for Scoping Reviews (PRISMAScR) guidelines [12,13]. Three databases were searched: PubMed, EMBASE, and Web of Science. The protocol was not published in advance (Additional Table S1 provides the completed PRISMA-ScR Checklist).

### 2.1. Eligibility Criteria

Eligible publications were those that addressed *Pneumocystis* infection or colonization in pregnant women. Publications were excluded if they referenced *Pneumocystis* infection in animals. No date or language limits were applied to the databases.

### 2.2. Search Strategy

We searched for peer-reviewed research using PubMed, Embase, and Web of Science databases to capture a broad range of study designs. The most recent search was executed in December 2024.

The search strategy was anchored on key terms related to pregnancy and *Pneumocystis* infection or colonization in the three databases (Table 1).

The search string was connected by Boolean operators. Duplicate references were removed to the extent possible, and the articles were screened for eligibility based on the established inclusion and exclusion criteria prior to data extraction.

### 2.3. Study Screening and Selection

The results from the literature search were imported into Zotero 6 (version 6.0.36, Corporation for Digital Scholarship, Vienna, VA, USA) with duplicates removed. Article titles and abstracts were independently reviewed for eligibility by the authors. Once references were determined to be potentially eligible, full articles were obtained for studies that appeared to meet the eligibility criteria based on title and abstract screening. Next, the full text was independently reviewed by two coders for eligibility and inclusion. Six authors (ICB, RS, YdA, PG, EC, and RM) assumed this role and completed the title/abstract and full-text screening. In both the abstract and full-text stages, disagreement was resolved by bringing the corresponding author (EJC) in for discussion if required.

### 2.4. Data Extraction and Synthesis

Data synthesis tables were developed a priori by two reviewers (ICB and RM). The tables consisted of predefined data items for extraction, including title, authors, and publication year. Tables were revised iteratively as needed while screening each of the articles included. Four reviewers (RS, YdA, PG, and EC) extracted data from the original search; a final reviewer then independently verified the data (VF). Any discrepancies were resolved by re-review of the study or discussion with the final reviewer (VF).

## 3. Results

**Figure 1:**
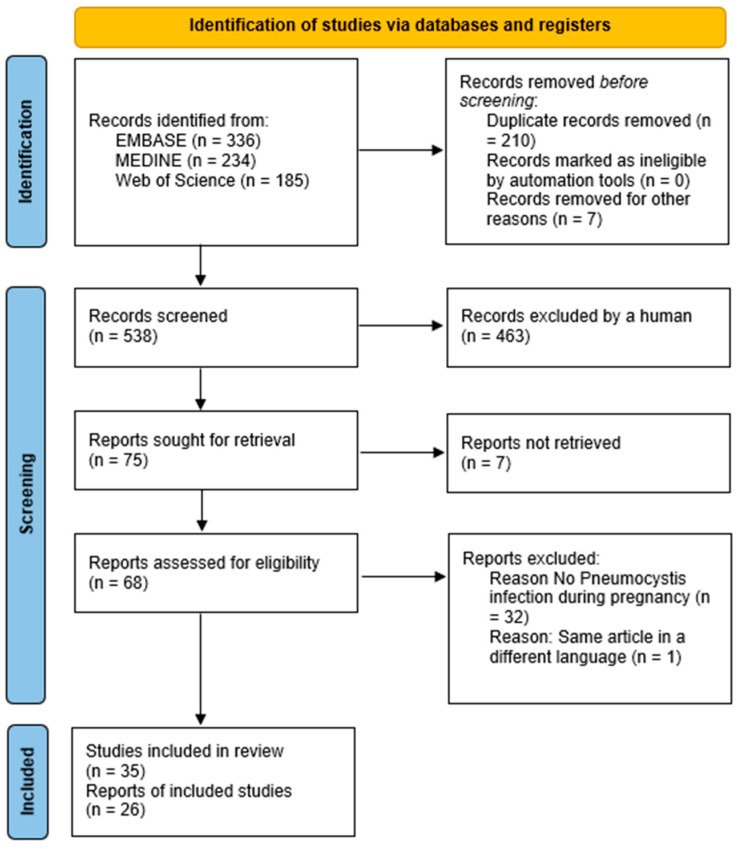
Flow chart of study selection and screening process.

The search yielded a total of 755 articles. After the duplicates were identified and removed, 538 unique articles were included (Figure 1). Title and abstract screening resulted in the exclusion of 463 articles, and the remaining 75 articles were assessed for eligibility based upon the objectives of this scoping review. Seven articles were unable to be obtained for full-text reading. After that, 33 articles were further excluded by full-text screening, resulting in a final total of 35 articles (Figure 1).

The reasons for the full-text exclusion phase were mainly since *P. jirovecii* infection did not occur during pregnancy or *Pneumocystis* was not included among the pathogens identified as the cause of infection during pregnancy. In one case, the article was excluded because it was duplicated in another language and reported a case of PcP in a stillborn child with scarce mother information [14,15]. Of these 35 articles, the reports of 26 were included in the study, while the remaining 9 reports were excluded because they reported cases of PCP that were already published in a summary article that was included [16,17,18,19,20,21,22,23,24,25].

At the end of the selection process, 26 articles were included. Among them, 19 articles were related to PCP during pregnancy, describing 94 cases (Table 2), while 7 articles reported data on *P. jirovecii* colonization (Table 3).

Concerning articles focusing on PCP during pregnancy, 8 of the 19 articles originated in Western Europe (42.1%), 3 (15.7%) in the USA, 3 (15.7%) in Japan, 2 (10.5%) in Russia, and 1 (5.2%) in China, India, and South Africa each. The newest study was published in 2023 and the oldest was published in 1985 [26,43].

Regarding *Pneumocystis* colonization, 4 of the 7 articles originated in South America (57.1%), 2 (28.5) in Western Europe, and 1 (14.2) in Russia. The oldest study was published in 2003 and the newest in 2022 [11,48].

Information available on PCP during pregnancy is limited in most articles to case descriptions, with no data on its incidence or frequency in the pregnant population (Table 2).

The demographic and clinical information provided in the PCP reports in pregnant women is heterogeneous and, in some cases, incomplete. In general, the characteristics of PCP cases in pregnant women with HIV infection do not differ from the presentation of AIDS-related PCP [25,40]. Both acute and subacute presentations have been described [25,34]. Usually, these patients have low CD4+ T cell counts, but PCP cases have also been described in pregnant women with HIV infection and normal CD4+ T cell counts [39]. Both forms of PCP, acute and subacute, have also been described in pregnant women without HIV infection [26,30]. The main clinical symptoms are dyspnea, which increases over time; nonproductive cough or cough that produces clear sputum; low-grade or no fever; malaise; and sometimes chest tightness or pain. The clinical onset could be insidious or acute and cause severe respiratory distress that could require mechanical ventilation within the first few days [26,34]. The clinical picture in individual patients is variable, and other infectious processes can present identically; therefore, a high index of suspicion is necessary in all cases.

On the other hand, information on *Pneumocystis* colonization comes from very few studies with differing results (Table 3).

Demographic and clinical information on carrier mothers is very limited or absent. Only the article by Vera et al. details the age of colonized mothers (28.7 ± 5.1 years) and non-colonized mothers (30.5 ± 5.9 years) [46]. This article also reports that 40% of colonized mothers had respiratory symptoms and as did 39.1% of non-colonized mothers but does not specify which type of symptoms [46]. The article by Vargas et al. indicates that the ages of the pregnant women studied ranged from 14 to 39 years but does not differentiate between colonized and non-colonized women [48]. In the article by Szydowicz et al., the ages of the pregnant women ranged from 18 to 43 years but the study did not specify age in relation to colonization [11]. In the remaining articles, no specific demographic or clinical information about mothers appears [9,44,47].

## 4. Discussion

The main objective of this review was to identify the existing knowledge about *Pneumocystis* infection or colonization in pregnant women.

Pregnancy has been suggested as a possible risk factor for asymptomatic carriage of *P. jirovecii* [49]. This is based on changes in the immune system that occur during pregnancy. The cell immune response with Th1 decreases, whereas the humoral response with Th2 increases proportionally, in addition to certain cytokines involved in the innate and humoral response, such as IL-4, IL-6, and IL-10 [46,50]. On the other hand, PCP has been described to have a more aggressive course during pregnancy, with increased morbidity and mortality. In the review by Ahmad et al. among 22 pregnant women with PCP, the mortality rate was 50%, which is higher than that usually reported for HIV-infected individuals with PCP, and 13 developed respiratory failure (59%) requiring mechanical ventilation [25].

Our results have been organized into two main sections: descriptions of PCP cases in pregnant women and colonization during pregnancy.

In this review, we have found that 75.5% of PCP reported cases were in HIV-positive pregnant women before the introduction of antiretroviral therapy [16,19,22,24,39,40,42]. Subsequently, another 28 cases of PCP have been reported in pregnant women with HIV infection, most of whom were women who were unaware of their infection or did not comply with antiretroviral treatment [28,34,35,36,37,38]. However, in the last 10 years, all published PCP cases in pregnant women, except one, occurred in HIV-negative women who suffered from hematologic malignancies or, in one case, lung cancer; none of them were undergoing chemoprophylaxis for PCP [26,27,30,31,32].

These data are consistent with epidemiological studies of hospital records showing a decrease in PCP cases among HIV-infected patients after the introduction of antiviral therapy, while PCP increases in other risk groups, such as patients with hematologic malignancies [51,52]. Although PCP during pregnancy in HIV-negative patients is very rare, it is notable that the mortality rate of PCP patients in the absence of HIV is higher (30 to 60%) than in patients with HIV infection (10 to 20%). Furthermore, PCP in HIV-negative patients has an abrupt onset consisting of respiratory failure and the patient can deteriorate rapidly. Therefore, appropriate prophylaxis is necessary to reduce the risk of PCP, even among HIV-negative pregnant women if they have other known causes of immunodepression [30,53].

*Pneumocytis* pneumonia is a clinical challenge during pregnancy because its prompt diagnosis and appropriate treatment are essential for a good prognosis. However, PCP is sometimes difficult to diagnose, as patients present nonspecific symptoms such as fever, dyspnea, and nonproductive cough. In pregnant women, doctors may be reluctant to perform chest radiographs due to the risk of fetal radiation. However, for the diagnosis of PCP, chest CT is more sensitive and superior as a diagnostic tool than chest radiography and it can be used in pregnant women [54]. The doses of fetal radiation from chest CT are very low, as this type of scan does not involve direct irradiation but rather results in exposure to scattered radiation. The maximum estimated conceptus radiation dose from chest CT is less than 1 mGy, with an average of 0.22 mGy. Therefore, it should be highlighted that physicians should not hesitate to perform chest CT examinations, even during pregnancy, when PCP is suspected because CT is safe [30,55].

The information available on *P. jirovecii* colonization during pregnancy is scarce and comes from a few studies, including a small number of case studies. The prevalence of colonization in studies using oropharyngeal or nasal lavage samples from pregnant women ranges from 5.4% in a study conducted in Peru to 46.5% in a study from Colombia [45,46].

These differences in prevalence between different countries have also been observed in studies conducted in different groups, such as subjects with cystic fibrosis, with prevalences of 2.5% in Brittany, France, and 31.6% in Seville, Spain, or subjects with chronic obstructive pulmonary disease (COPD), with prevalence ranging from 7.9% in Iran to 55% in Spain [56,57,58]. These differences could be in part due to real variations caused by climatic factors, but also due to the different clinical contexts, sampling methods, laboratory reagents, or technical strategies used for DNA extraction, amplification, or analysis of results [7].

However, the two studies with comparable methodology focusing on *P. jirovecii* research in the placenta of mothers show similar results, 38.4% in one case and 40% in the other, although they come from different geographical areas [9,44]. It should be noted that the demonstration of the presence of *P. jirovecii* in the placenta confirms its ability to be transmitted vertically. Furthermore, the high frequency found in both studies suggests that this form of transmission is not an occasional phenomenon. Indeed, horizontal airborne transmission does not appear to be the only modality of interhost transmission of some *Pneumocystis* species. Evidence of vertical transmission by the transplacental route has been found in other mammals, such as rabbits (Oryctolagus cuniculus) [59,60]. However, in utero transmissibility of *Pneumocystis* does not appear to be a characteristic common to all species. Transplacental transmission has also been explored in rats and mice, but it does not appear to occur in these rodents, which have a different hemotrichorial placenta from rabbits or human placenta [61,62,63]. This route of transmission could allow the fungus to spread while “protected” from environmental hazards. But the consequences for the host are largely unknown, supporting the interest in exploring the mode of in utero transmission of *P. jirovecii* and the potential role of this fungus in maternal–fetal health

In both cases of PCP in pregnant women and in cases of colonization, the information available in the articles on the effect of maternal infection in newborns is very scarce and is absent in most articles. Regarding mothers who developed PCP during pregnancy, three articles report the death of the fetus, another describes an abortion, and another reports a premature birth [24,28,31,38,43]. Regarding the articles on *Pneumocystis* colonization in pregnant women, one of them reports the presence of PCP in the newborn, and two others describe the presence of primary infection but without specifying if they developed PCP [11,46,47]. In one of these articles, primary infection was associated with the development of bronchopulmonary dysplasia in newborns [11]. Finally, in the Montes-Cano article, all of the cases studied corresponded to intrauterine fetal deaths [9]. Although the available information is poor, the available data suggest that *P. jirovecii* infection or colonization during pregnancy may have repercussions on maternal and child health that have been ignored until now and deserve to be considered and studied.

Despite the interesting information provided by this scoping review on PCP and *Pneumocystis* colonization in pregnant women, several limitations must be recognized. These limitations highlight the challenges encountered during the research process and the need for future studies to address existing gaps.

One of the main limitations of this study is the heterogeneity of the included research articles. The reviewed studies varied significantly in terms of sample size, study design, diagnostic methods, and patient populations. This variability limits the ability to draw definitive conclusions and compare results in different studies. Furthermore, due to differences in the type of biological samples and laboratory techniques used for the detection of *P. jirovecii*, the reported prevalence rates of colonization may not be entirely consistent. Variations in DNA extraction, amplification techniques, and PCR protocols may have influenced detection rates, leading to discrepancies in findings between different geographical regions and study settings.

The impact of *Pneumocystis* infection or colonization on maternal and neonatal outcomes is not adequately explored. Most studies focused on the presence of *P. jirovecii* in maternal and fetal biological samples but did not provide detailed follow-up data on neonatal health outcomes, such as respiratory complications or long-term morbidity. Prospective cohort studies with standardized data collection protocols would be more beneficial in elucidating the true burden and clinical implications of *Pneumocystis* colonization and infection during pregnancy.

Geographical variability in the included studies also poses a major limitation. Most of the data were derived from a limited number of countries, with a predominance of studies conducted in Europe and South America. This geographic imbalance may not accurately reflect global trends in *P. jirovecii* infection among pregnant women, particularly in low-resource settings where access to healthcare and diagnostic capabilities differ significantly [64]. Further research in diverse populations, including those in Africa and Asia, is essential to provide a more comprehensive understanding of the epidemiology of *Pneumocystis* in pregnancy.

Additionally, the role of potential confounding factors, such as co-infections, socioeconomic status, and environmental exposure, was not systematically addressed in the studies. These factors could influence the risk of colonization and infection of *Pneumocystis*, but their impact remains largely unexplored in the literature [65]. Future studies should consider a multifactorial approach that accounts for these variables to improve the accuracy and applicability of the findings.

Although this study provides important information on PCP and *Pneumocystis* colonization in pregnancy, the identified limitations underscore the need for more research. Standardized diagnostic methods, comprehensive clinical data collection, and a broader geographic representation will be critical to advance our understanding of this opportunistic pathogen in the context of maternal and neonatal health.

## 5. Conclusions

In this context, a thorough understanding of *Pneumocystis* in the pregnant population is essential for clinicians and researchers. The findings of this scoping review are expected to raise awareness of the need for HIV screening in pregnant women and the need to include PCP in the diagnosis of pregnant women with pneumonia. Furthermore, we hope that it will encourage further research on the effect of *P. jirovecii* infection/colonization on both maternal and child health, ultimately improving the outcomes for affected mothers and their offspring. Additionally, this work will contribute to the growing body of research on opportunistic infections in pregnancy, highlighting the need for increased awareness and specialized care in this vulnerable group.

Moreover, it highlights critical research gaps, particularly in the areas of vertical transmission and its impact on neonatal respiratory health, providing a foundation for future studies aimed at improving results in this vulnerable population.

## Figures and Tables

**Table 1:** Search strategy used in each database.

Database	Search Strategy
PubMed	(“pneumonia, *pneumocystis*” [MeSH Terms] OR (“pneumonia” [All Fields] AND “*pneumocystis*” [All Fields]) OR “*pneumocystis* pneumonia” [All Fields] OR “*pneumocystis*” [All Fields] OR “*pneumocystis*” [MeSH Terms]) AND (“pregnancy” [MeSH Terms] OR “pregnancy” [All Fields] OR “vertical transmission” [All Fields])
EMBASE	(‘pneumocystosis’/exp OR ‘pneumocystosis’) AND (‘pregnancy’/exp OR ‘pregnancy’)
Web of Science	Pneumocystis and Pregnancy (Articles + case reports) excluded Review articles, Awarded Grant and Other

**Table 2:** *Pneumocystis* pneumonia in pregnant women.

Authors [Ref]	Year	Country	Cases	Immunosuppression	Offspring
Hu et al. [26]	2023	China	1	Non-Small-Cell Lung Cancer	NAD
Onishi et al. [27]	2022	Japan	3	Lymphoma	NAD
Fritzsche et al. [28]	2021	Germany	1	HIV	Premature
Trier-Morch et al. [29]	2017	Denmark	1	No apparent	NAD
Fukutani et al. [30]	2017	Japan	2	Hodgkin’s lymphoma B-cell lymphoma	Emergent cesarean section Newborn; uneventful
Bazhenov et al. [31]	2016	Netherlands	4	Acute leukemia	1 miscarriage
Galstyan et al. [32]	2015	Russia	1	Leukemia	NAD
Tamaki et al. [33]	2011	Japan	1	HTLV-I	NAD
Parisaei et al. [34]	2010	UK	1	HIV	NAD
Bera E. [35]	2009	South Africa	2	HIV	NAD
McNally et al. [36]	2005	UK	1	HIV	*Pneumocystis* infection
Gervasoni et al. [37]	2003	Italy	1	HIV	NAD
Ahmad et al. [25]	2001	USA	22	HIV	NAD
Kumar et al. [38]	1997	India	5	HIV	Fetal Death
Deresiewicz et al. [39]	1996	USA	1	No	NAD
Stratton et al. [40]	1992	USA	35	HIV	NAD
Bongain et al. [41]	1992	France	2	NAD	NAD
Constantopoulos et al. [42]	1987	France	9	HIV	NAD
Kurennaia et al. [43]	1985	Russia	1	NAD	Fetal death

NAD: not available data.

**Table 3:** *Pneumocystis* colonization in pregnant women.

Authors [Ref]	Year	Country	Cases/Population	Immunosuppression	Mother Sample	Offspring
Szydłowicz et al. [11]	2022	Poland	7/31 (22.5%)	Non-immunosuppressed	Oral washes	8/56
Vargas S. [44]	2022	Chile	35/91 (38.4%)	NAD	Placenta	NAD
García et al. [45]	2020	Perú	5/92 (5.4%)	Non-immunosuppressed	Oropharyngeal washes, nasal swabs	0/5
Vera et al. [46]	2017	Colombia	20/43 (46.5%)	Non-immunosuppressed	Nasopharyngeal swabs	12/20
Samitova et al. [47]	2016	Russia	1/1	NAD	Placenta	1 PcP
Montes-Cano et al. [9]	2009	Spain	8/20 (40%)	Non-immunosuppressed	Placenta	5/8
Vargas et al. [48]	2003	Chile	5/33 (15.1%)	Non-immunosuppressed	Nasal swab	NAD

NAD: not available data.

## Data Availability

The original contributions presented in this study are included in the article/Supplementary Material. Further inquiries can be directed to the corresponding authors.

## Supplementary Materials

The following supporting information can be downloaded at https://www.mdpi.com/article/10.3390/jof11040327/s1: Table S1: Preferred Reporting Items for Systematic reviews and Meta-Analyses extension for Scoping Reviews (PRISMA-ScR) Checklist [13].
